# The mechanism by which piR-000699 targets SLC39A14 regulates ferroptosis in aging myocardial ischemia/reperfusion injury: piR-000699 targeting *SLC39A14* regulates ferroptosis in myocardial I/R injury

**DOI:** 10.3724/abbs.2024024

**Published:** 2024-03-04

**Authors:** Hongyang Chi, Yue’e Chai, Lingju Ma, Yichen Wang, Qianqian Wu, Lexin Wang, Junjie Zhai, Fufun Ma, Yancheng Tian, Ning Qi, Jianhong Peng, Youjuan Fu, Xiaoling Yang, Hui Huang, Shengchao Ma

**Affiliations:** 1 NHC Key Laboratory of Metabolic Cardiovascular Diseases Research Ningxia Medical University Yinchuan 750004 China; 2 Ningxia Key Laboratory of Vascular Injury and Repair Research Ningxia Medical University Yinchuan 750004 China; 3 College of Pharmacy Guizhou Medical University Guiyang 561113 China; 4 Department of Geriatrics and Special Needs Medicine General Hospital of Ningxia Medical University Yinchuan 750004 China; 5 School of Laboratory Medicine Ningxia Medical University Yinchuan 750004 China

**Keywords:** piR-000699, *SLC39A14*, ferroptosis, aging myocardial ischemia/reperfusion injury

## Abstract

Myocardial ischemia/reperfusion (I/R) injury is a classic type of cardiovascular disease characterized by injury to cardiomyocytes leading to different types of cell death. The degree of irreversible myocardial damage is closely related to age, and ferroptosis is involved in cardiomyocyte damage. However, the mechanisms underlying ferroptosis regulation in aging myocardial I/R injury are still unclear. The present study aims to explore the underlying mechanism of piRNA regulation in ferroptosis. Using left anterior descending coronary artery ligation in an aging rat model and a D-galactose-induced rat cardiomyocyte line (H9C2) to construct an aging cardiomyocyte model, we investigate whether ferroptosis occurs after reperfusion injury *in vitro* and *in vivo*. This study focuses on the upregulation of piR-000699 after hypoxia/reoxygenation treatment in aging cardiomyocytes by observing hypoxia/reoxygenation (H/R) injury indicators and ferroptosis-related indicators and clarifying the role of piR-000699 in H/R injury caused by ferroptosis in aging cardiomyocytes. Bioinformatics analysis reveals that *SLC39A14* is a gene that binds to piR-000699. Our data show that ferroptosis plays an important role in I/R injury both *in vivo* and *in vitro*. Furthermore, the results show the potential role of piR-000699 in regulating SLC39A14 in ferroptosis in aging cardiomyocytes under hypoxia/reoxygenation conditions. Together, our results reveal that the mechanism by which piR-000699 binds to *SLC39A14* regulates ferroptosis in aging myocardial I/R injury.

## Introduction

Ischemia-reperfusion (I/R) is a prominent pathological process that occurs in numerous organs and diseases [1]. Myocardial I/R injury represents an inevitable risk event for acute myocardial infarction, resulting in morbidity and mortality among elderly patients worldwide [2]. Although significant advances have been made in the treatment of I/R injuries, the development of cardioprotective therapeutics remains a formidable challenge, with the severity of damage closely associated with age [3]. However, the underlying mechanism remains largely elusive; hence, it is imperative to explore novel therapeutic targets for managing aged myocardial I/R injury.

Ferroptosis, a recently defined iron-dependent form of cell death, is characterized by the generation of reactive oxygen species (ROS) and lipid peroxidation [4]. In contrast to apoptosis, necrosis, and autophagy, which are distinct in morphology, biochemistry, and genetics [5], ferroptosis primarily arises due to dysregulated iron metabolism leading to excessive ROS production and lipid peroxide accumulation, culminating in cellular death [6]. Numerous studies have reported associations between ferroptosis and pathophysiological processes implicated in various diseases, including cancer progression, tissue or organ damage, stroke occurrence, I/R injury and renal degeneration [7]. A previous study confirmed that proteins related to ferroptosis are involved in the process of myocardial ischaemia-reperfusion injury, which is supported by evidence suggesting that selective inhibition of ferroptosis within cardiomyocytes may alleviate myocardial damage [8]. Gao *et al*. [9] reported that the overexpression of growth and differentiation factor 15 significantly inhibits MIRI while improving cardiac function by alleviating MIRI-induced ferroptotic cell death. However, the regulatory mechanisms governing ferroptosis during MIRI remain unknown, particularly concerning aging-related myocardial I/R injury.

P-element-induced wimpy testis (PIWI)-interacting RNAs (piRNAs) are recently discovered small noncoding RNAs found in germ and somatic cells that consist of 24‒31 nucleotides (nt) with a preference for adenosine at the 10th position or a 5′-terminal uridine [ 10, 11] . These piRNAs lack distinct secondary structure motifs, yet they exert regulatory control over gene expression in somatic cells through various mechanisms, such as transposon silencing, epigenetic programming, DNA rearrangement, mRNA turnover, and translation [ 12, 13] . Alterations in the expressions of numerous piRNAs have been associated with myocardial hypertrophy [14]. In progenitor cells within the mesoderm and cardiomyocytes alone, a total of 447 piRNA transcripts were identified; among them, 218 were detected in the mesoderm, while 171 were detected in cardiomyocytes. This suggests that genes carrying piRNA transcripts within cardiac tissue play crucial roles in key biological processes involving the heart [15]. However, despite presenting an intriguing new avenue for basic and translational research on aging myocardial I/R injury regulation via ferroptosis induction by piRNAs, their specific functions and underlying mechanisms remain largely unknown.

In the present study, we revealed that one particular piRNA known as piR-000699 plays a pivotal role in both initiating and advancing aging myocardial I/R injury. Furthermore, we identified *SLC39A14* as an important target gene regulated by piR-000699 that modulates H/R injury specifically within aging cardiomyocytes induced by ferroptosis. Finally, our investigation further elucidated the precise role that played by both piR-000699 itself and its functional targetting *SLC39A14* in regulating ferroptosis in aging cardiomyocytes.

## Materials and Methods

### Animals and experimental protocols

Male, aged Sprague-Dawley rats weighing between 600 g and 700 g (22‒24 months) were purchased from Speifu Biotechnology Co., Ltd (Beijing, China). The aged rats were maintained under standard conditions (20‒26°C, 40%‒70% humidity, with a 12 h light/ 12 h dark cycle) and provided a standard diet and water ad libitum. The aged rats were intraperitoneally injected with pentobarbital (45 mg/kg body weight). After anaesthesia, there was no response in the clip toe test, and the trachea was intubated. The ventilator parameters were configured to synchronize with the respiratory rate of the anaesthetized mice. Then, the endotracheal tube was attached to the ventilator. Before and after the operation, a physiological signal collection and processing system was utilized to record the rat′s electrocardiogram. The left anterior descending (LAD) coronary artery was ligated with a 7-0 silk suture below the left atrial appendage and the left edge of the pulmonary cone for 30 min to induce myocardial ischemia. After 30 min of ischemia, the silk thread was loosened for reperfusion for 180 min to establish the I/R model. Sham-operated rats underwent all surgical procedures except for the ligation step [16]. Finally, the chest and skin were closed, and the respiratory machine was kept on to ventilate the rat with oxygen until it woke up. Rat cardiac function was evaluated using a high-resolution echocardiography imaging system (VINNO Technology, Suzhou, China). Hearts were imaged two-dimensionally in long-axis views, and cardiac function, including heart rate, LVIDd, and LVIDs, was assessed. FS was determined as follow: (LVIDd−LVIDs)/LVIDd×100%. EF was determined by the formula: [end-diastolic volume (EDV)−end-systolic volume (ESV)]/EDV×100%.

### Cell culture

H9C2 cells were cultured according to our previously reported protocol. The environment of the anaerobic chamber was maintained as described previously; then, the aged cardiomyocytes were subjected to hypoxia (95% N _2_, 5% CO _2_) for 180 min followed by 120 min of reoxygenation to establish the hypoxia/reoxygenation (H/R) model. Cell grouping: i) normoxia group: aging myocardial cells were cultivated at 37°C under normal oxygen conditions; ii) H/R group: nitrogen was used to induce hypoxia in the cell culture incubator, and aged myocardial cells were subjected to hypoxia for 5 h before being transferred to a normal cell culture incubator for reoxygenation for 3 h; iii) H/R+mimics NC group; iv) H/R+ Inhibitor-NC group; v) H/R+piR-000699 mimics group; and vi) H/R+piR-000699 inhibitor group.

### HE staining

The rats were anaesthetized via sevoflurane inhalation. The rat myocardium was carefully stripped, and ophthalmic scissors were used to cut the rat myocardium. The rat myocardium was fixed with cold phosphate buffer solution (PBS), embedded in optical coherence tomography and frozen at ‒20°C for 30 min. The thickness of the section was 10 μm, and the section was maintained at ‒80°C. Then, two slides were selected from each sample and stained. The samples were subjected to gradient alcohol dehydration, haematoxylin and eosin (HE) staining, haematoxylin staining for 3 min, ethanol fractionation with hydrochloric acid for 10 s, eosin staining for 3 min, conventional dehydration, transparency, blocking and light microscopy to observe changes in the myocardial cells. The morphology and structure of normal and abnormal myocardial cells on each slide were investigated under a light microscope (Leica, Wetzlar, Germany) at 200× magnification.

### Masson staining

The frozen sections were frozen at room temperature, soaked in Masson solution at room temperature overnight, and washed with tap water for 30 s until the yellow color on the tissues faded. The Masson B solution and Masson C solution were mixed in equal volumes, the slices were dipped into the mixed solution for 1 min, and the slices were washed slightly with running water. After differentiation with 1% hydrochloric acid and ethanol (concentrated hydrochloric acid:absolute ethanol=1:100), the slices were lightly washed with tap water for approximately 1 min, drained and soaked in Masson D solution for 3 min, and then washed with tap water for approximately 20 s until the water flowing on the slices became colorless. The water (the tablet can not be dried) was removed, and the slices were soaked in Masson’s solution for approximately 1 min. After the slices were slightly drained of Masson’s solution, they were directly dyed in Masson’s solution for 2‒30 s without washing. The slices were rinsed and differentiated in three consecutive cylinders of 1% glacial acetic acid aqueous solution for approximately 7 s per cylinder. Then, the samples were dehydrated for approximately 3 s, 5 s, and 5 s successively through three consecutive cylinders of anhydrous ethanol. The slides were then washed with xylene for 5 min and sealed with neutral gum.

### Transfection of aged cardiomyocytes with the piR-000699 mimic, inhibitor and SLC39A14 inhibitor

The piR-000699 mimic, piR-000699 inhibitor, and SLC39A14 inhibitor ( Table 1) were obtained from GenePharma (Shanghai, China). The cells were transiently transfected into aged cardiomyocytes using the INVI DNA RNA Transfection Reagent (Invitrogen, Carlsbad, USA) following the manufacturer′s instructions. The transfection efficiency was measured by qRT‐PCR and western blot, analysis after which the cells were collected for subsequent analysis.

**Table:** **Table 1** siRNA sequences used in this study

Name	Sequences (5′→3′)
piR-000699 mimics	Sense	CGUUGGAUCGAUGUGGUGCUGCCGCGU
	Antisense	GCGGCAGCACCACAUCGAUCCAACGUU
piR-000699 inhibitor	Sense	ACGCGGCAGCACCACAUCGAUCCAACG
SLC39A14 inhibitor	Sense	GCAGUCACUUCUCUGCAAATT
	Antisense	UUUGCAGAGAAGUGACUGCTT

### Enzyme-linked immunosorbent assay (ELISA)

According to the instructions of the ELISA kit (Shanghai Jianglai Industrial Limited Co. Ltd, Shanghai, China), the lactic dehydrogenase (LDH) and creative kinase isoenzyme MB (CK-MB) levels in the serum or cell supernatant were measured. First, 100 μL of sample or standard working solution was added to the corresponding plate wells and incubated for 90 min at 37°C. After incubation, the liquid inside the plate was aspirated, and 100 μL of LDH or CK-MB biotinylated antibody working solution was added. Then, the plate was incubated at 37°C for 60 min. After the incubation, the working solution in the plate was discarded, and the plate was washed 3 times. Thereafter, 100 μL of HRPase conjugate working solution was added to the plate and incubated for 30 min at 37°C. After the incubation, the liquid in the plate was aspirated, and the plate was washed 5 times. Then, 90 μL of substrate solution was added, and the plate was incubated for 15 min at 37°C. Finally, 50 μL of stop solution was added to each well. After the reaction was terminated, the absorbance was read at a wavelength of 450 nm within 5 min.

### Measurement of the aged myocardial infarct size

The 2,3,5-triphenyltetrazolium chloride (TTC; Sigma Aldrich, St Louis, USA) staining method was used to measure the myocardial infarction size in the isolated hearts of the rats in each group. After the experiment, the heart was removed and placed in a refrigerator at –20°C for 5 min. Then, the whole heart was sliced at a thickness of 2–3 mm. The sections were placed in a 2% TTC solution dissolved in 0.1 M PBS and kept in a water bath at 37°C for 15 min in the dark. Afterwards, the samples were fixed with 10% paraformaldehyde for 30 min before image acquisition. Normal areas were stained red by TTC, and infarct areas (IAs) were stained white. The IA and AAR were analyzed using Image-Pro Plus 6.0. The infarct size was calculated as the percentage of IA over AAR (IA/AAR×100%).

### Quantitative real-time polymerase chain reaction (qRT-PCR)

First-strand cDNA was synthesized from total RNA using the Revert Aid First-Strand cDNA Synthesis kit (Thermo Scientific, Waltham, USA). TB Green™ Premix Ex Taq™ (Takara, Dalian, China) was used for amplification according to the manufacturer’s instructions in a QuantStudio5 real-time PCR detection system (Thermo Scientific). *β-Actin* and *U6* (MQP-0201; Guangzhou Ruibo Biotechnology Co., LTD, Guangzhou, China) were used as normalization controls for mRNAs and piRNAs, respectively. The specific primers used for piR-000699 were the Bulge-Loop™ piRNA primer (RiboBio, Guangzhou, China). The primer sequences for *TFR1*, *STEAP3*, *SLC39A14* and *ACSL6* are listed in Table 2, which are obtained from Sangon Biotech (Shanghai, China). The relative expression of mRNAs and piRNAs was analyzed using the 2 ^‒ΔΔct^ method and normalized to the endogenous control.

**Table:** **Table 2** Sequences of primers used in this study

Name	Sequences (5′→3′)
*TFR1*-F	GGTTCGTACAGCAGCAGAGGTG
*TFR1*-R	TCCACGAGCAGAATACAGCCATTG
*STEAP3*-F	GCAGCACCGCAAGCAGATCG
*STEAP3*-R	TCCATCCGCCAGACTTCTTCCTC
*SLC39A14*-F	TCCTGGCTGGCAGTCACTTCTC
*SLC39A14*-R	TTCTCATCCTCCTGGCACACCTC
*ACSL6*-F	AGCGAATGGTGCAGTCTGTTGTC
*ACSL6*-R	CGTCTGAGAGGAGGCGGATGTC
*β-actin*-F	TGTCACCAACTGGGACGATA
*β-actin*-R	GGGGTGTTGAAGGTCTCAAA

### Western blot analysis

Equal amounts of protein (30 μg) were subjected to electrophoretic separation via sodium dodecyl sulfate-polyacrylamide gel electrophoresis gel (SDS-PAGE) and subsequently transferred onto a polyvinylidene difluoride (PVDF) membrane (Millipore, Billerica, USA). After blocking with nonfat milk in PBST for 2 h at room temperature, the PVDF membranes were incubated with primary antibodies at 4°C overnight. Information about these antibodies is shown in Table 3. After washing with PBST, the membranes were incubated with secondary antibodies at room temperature for 2 h. The membranes were developed with an enhanced chemiluminescence solution (Invitrogen) and analyzed using Image Lab 5.1 software.

**Table:** **Table 3** Information of antibodies used in this study

Antibodies	Manufacturing company / code
TFR	Thermo Fisher / 13-6800
GPX4	Thermo Fisher / PA5-18545
ACSL4	Thermo Fisher / PA5-27137
SLC39A14	Thermo Fisher / AZT-024-200UL

### Iron assay

Iron concentrations in the myocardial tissue and cells were measured using an Iron Assay kit (Nanjing Jiancheng Bioengineering Institute, Nanjing, China) and a Cell Iron Content Assay kit (Solarbio, Beijing, China), respectively, according to the manufacturer’s instructions.

### MDA assay

Malondialdehyde (MDA) levels in the myocardial tissue and cells were measured using an MDA assay kit (BestBio, Shanghai, China) according to the manufacturer’s instructions.

### Dual-luciferase reporter assay

A luciferase reporter assay was utilized to confirm the relationship between the piRNAs and the target genes. The 3′UTR sequence of *SLC39A14* mRNA was chemically synthesized and introduced into a luciferase reporter vector to construct wild-type (WT) luciferase reporter plasmids. The seed region of piR-000699 in the 3′-UTR of *SLC39A14* was mutated to construct mutant (Mut) luciferase reporter plasmids (GeneChem, Shanghai, China). H9C2 cells were cotransfected with luciferase reporter plasmids and piRNA mimics after they reached 80% confluence by using Lipofectamine 2000 (Thermo Scientific) according to the manufacturer’s instructions. Forty-eight hours later, the cells were collected, and dual-luciferase activity was measured using a Dual-luciferase reporter assay (Promega, Madison, USA) according to the manufacturer’s instructions.

### Statistical analysis

The data are expressed as the mean±SD from at least three independent experiments. The results were analyzed with GraphPad Prism 8.0 software. One-way ANOVA, Student-Newman-Keul’s test (comparisons between multiple groups), or unpaired Student’s *t* test (between two groups) was used as appropriate. A value of *P*<0.05 was considered statistically significant.

## Results

### Establishment of aging myocardial I/R injury models *in vivo* and *in vitro*

**Figure 1:**
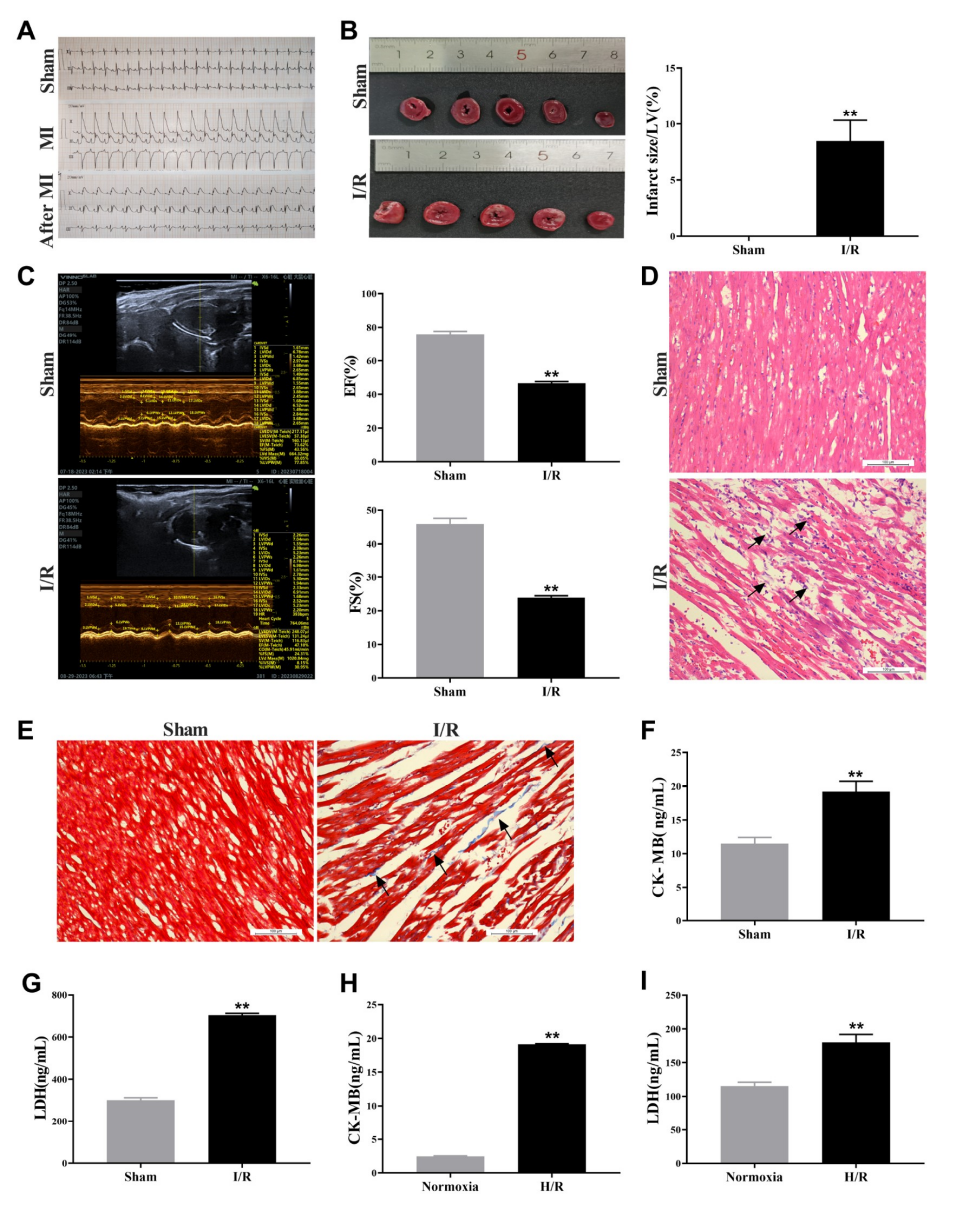
Figure 1 Establishment of aging myocardial ischemia/reperfusion injury models *in vivo* and *in vitro* (A) Heart electrical activity was detected using ECG. Left anterior descending artery ligation/reperfusion surgery was performed on rats from different groups to construct the myocardial I/R injury model. The ECG data of the rats were examined prior to and during MI and following MI injury. (B) Representative heart sections stained with Evans blue and 2,3,5-triphenyltetrazolium chloride (TTC) showing the infarct size in rats from the sham surgery and I/R surgery groups. Right panel, quantification of the infarct size in each group. Normal areas were stained with Evans blue, TTC staining (red) indicated the areas at risk (AAR), and white indicated the infarct areas (IAs). (C) Rat cardiac function was evaluated using a high-resolution echocardiography imaging system. Hearts were imaged two-dimensionally in long-axis views, and heart function was represented as the LV ejection fraction (EF) and fractional shortening (FS). (D) The results of HE staining at 200× for the sham and I/R groups. (E) The results of Masson staining of the myocardium in the I/R group and sham surgery group. (F,G) Serum levels of the myocardial cell death markers CK-MB and LDH determined by ELISA in the sham and I/R groups of rats. (H,I) Serum levels of the myocardial cell death markers CK-MB and LDH in H9C2 cells in the normoxia and H/R groups, as determined by ELISA. The results are presented as the mean±SD. n=3‒5. ** P<0.01 vs the sham/normoxia group. I/R, ischemia/reperfusion; ECG, electrocardiogram; MI, myocardial ischemia; LDH, lactate dehydrogenase.

To assess the models of aging myocardial I/R injury, we employed electrocardiography and echocardiography to evaluate the cardiac function of male SD rats following a 3-h period of myocardial I/R. In comparison to the sham group, the myocardial ischaemia (MI) group exhibited a significantly elevated ST segment on ECG, whereas the after MI group displayed a lower ST segment compared to that in the MI group ( Figure 1A). The ejection fraction and short-axis shortening rate of the left ventricle in the I/R group were lower than those in the sham group ( Figure 1C). TTC staining revealed distinct white infarct areas in heart slices from the I/R group, with a greater ratio of myocardial infarction size to left ventricular volume observed in the I/R group than in the sham group ( Figure 1B). HE staining revealed swollen, disordered, and loosely arranged myocardial cells accompanied by inflammatory cell infiltration in the I/R group ( Figure 1D). Masson staining revealed an increase in the presence of blue collagen fibres within the I/R group but fewer fibres within the Sham group ( Figure 1E). These findings suggested significant increases in LDH and CK-MB levels following a reperfusion period of three hours ( Figure 1F,G). Serum CK-MB and LDH levels were subsequently measured, revealing greater values for both parameters in the H/R groups than in the normoxia groups ( Figure 1H,I).

### Ferroptosis plays an important role in I/R injury *in vivo* and *in vitro*

**Figure 2:**
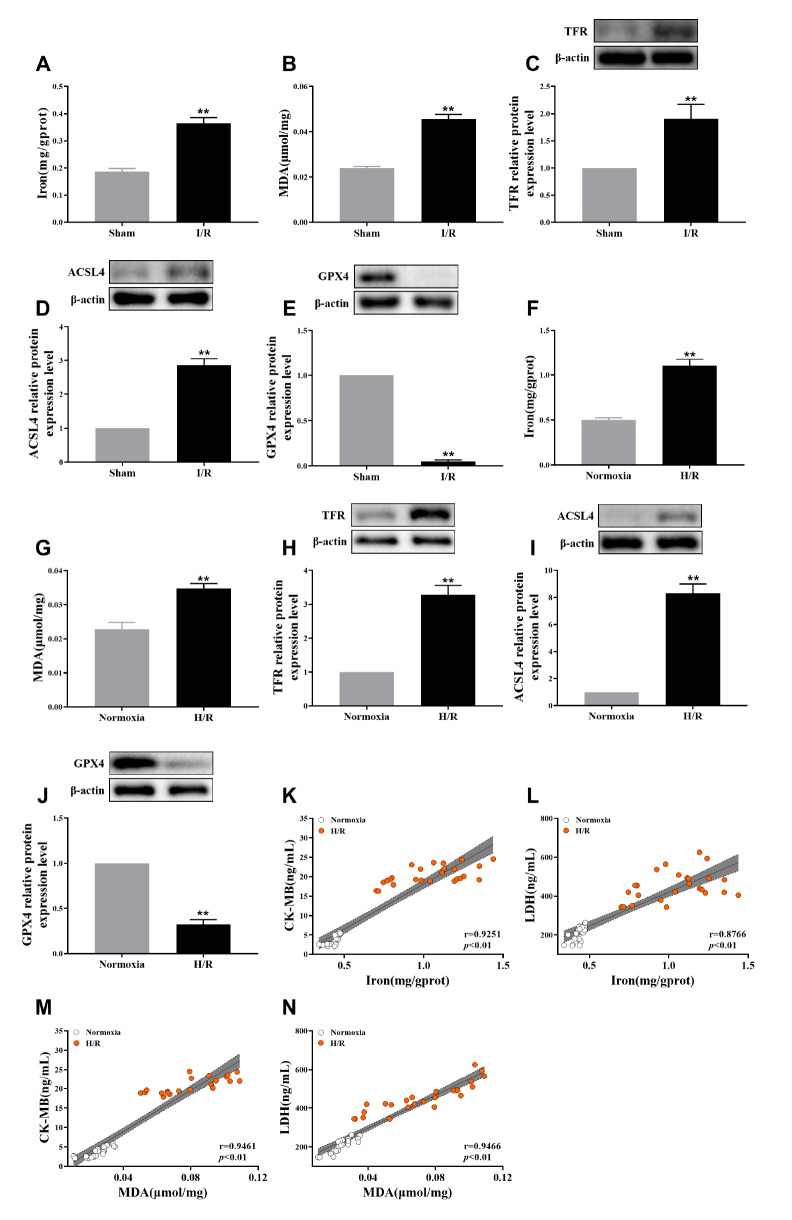
Figure 2 Ferroptosis plays an important role in I/R injury (A) The iron concentration in myocardial tissue was measured in the I/R group and sham surgery group. (B) Levels of the lipid peroxidation product MDA in heart tissues from the I/R group and sham surgery group. (C‒E) Western blot analysis was used the ferroptosis marker proteins of TFR, ACSL4 and GPX4 in the heart tissues of rats subjected to I/R surgery. (F) Iron concentrations in H9C2 cells in the normoxia and H/R groups were measured. (G) Levels of the lipid peroxidation products MDA in H9C2 cells in the normoxia group and H/R group. (H‒J) Western blot analysis of the ferroptosis marker proteins TFR, ACSL4 and GPX4 in H9C2 cells from the normoxia group and H/R group. (K) The correlation between serum CK-MB and iron in rats. (L) The correlation between serum LDH and iron in H9C2 cells. (M) The correlation between serum CK-MB and MDA in H9C2 cells. (N) The correlation between serum LDH and MDA in H9C2 cells. The results are presented as the mean±SD. n=3‒5. ** P<0.01 vs the sham/normocia group.

To further explore the potential role of ferroptosis in aging myocardial I/R injury, we utilized iron and MDA assay kits to measure the levels of iron and MDA in both the I/R group and sham surgery group. Our findings confirmed that the serum iron and MDA levels were significantly greater in the I/R group than in the sham group ( Figure 2A,B). Subsequently, western blot analysis was conducted to detect the key ferroptosis marker proteins TFR, ACSL4, and GPX4 in rat heart tissues subjected to I/R surgery. The results revealed that TFR and ACSL4 protein expression were increased within the myocardial tissue of the I/R group ( Figure 2C,D), while GPX4 expression was decreased ( Figure 2E). Furthermore, these indices were also assessed in H9C2 cells from both the normoxia and H/R groups and demonstrated changes consistent with those observed *in vivo* ( Figure 2F‒J). Additionally, Pearson correlation analysis was performed on myocardial enzyme levels as well as ferroptosis indicators detected across all groups. The results indicated a positive correlation between aging myocardial I/R injury markers both *in vivo* and *in vitro* and ferroptosis indicators ( Figure 2K‒N). Collectively, these findings strongly suggest that ferroptosis may serve as a crucial mechanism underlying I/R injury in aging myocardial cells.

### piR-000699 participates in aging myocardial I/R injury caused by ferroptosis

**Figure 3:**
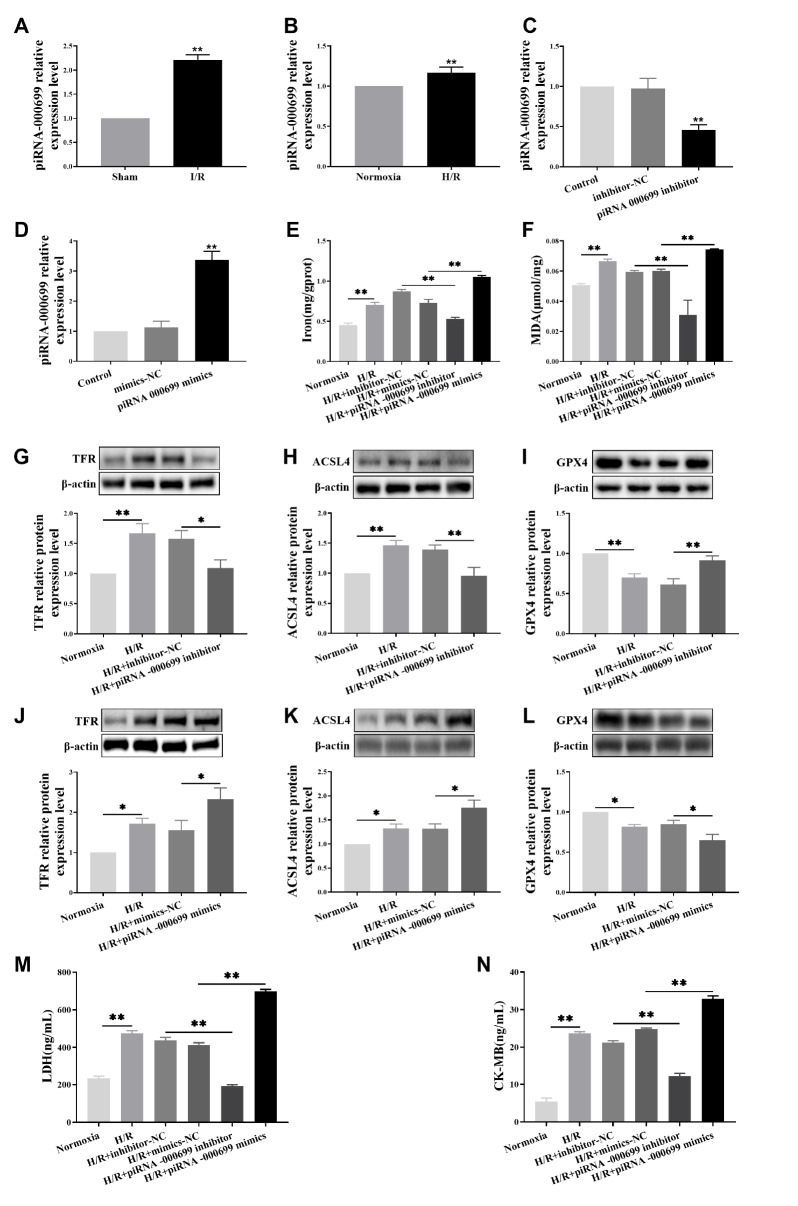
Figure 3 piR-000699 participates in aging myocardial I/R injury caused by ferroptosis (A) qRT-PCR was used to measure the expression of piR-000699 mRNA in the heart tissues of rats subjected to I/R surgery. (B) qRT-PCR was used to measure the expression of piR-000699 mRNA in H9C2 cells after they were subjected to H/R. (C) qRT-PCR was used to measure the expression of piR-000699 mRNA in H9C2 cells after the cells were treated with the piR-000699 inhibitor. (D) qRT-PCR was used to measure the expression of piR-000699 mRNA in H9C2 cells after the cells were treated with piR-000699 mimics. (E) Iron concentrations in H9C2 cells were measured. (F) The levels of the lipid peroxidation products MDA in H9C2 cells were measured. (G‒I) Western blot analysis was used to measure the ferroptosis marker proteins TFR, ACSL4 and GPX4 in H9C2 cells treated with the piR-000699 inhibitor. (J‒L) Western blot analysis was used to measure the ferroptosis marker proteins TFR, ACSL4 and GPX4 in H9C2 cells treated with piR-000699 mimics. (M,N) Serum levels of the myocardial cell death markers CK-MB and LDH determined by ELISA in H9C2 cells. The results are presented as the mean±SD. n=3‒5. ** P<0.01 vs the normocia group / H/R+mimics NC group / H/R+Inhibitor-NC group.

To investigate the potential involvement of piR-000699 in aging-related myocardial I/R injury, we initially assessed the expression levels of piR-000699 in rat heart tissues subjected to I/R surgery. Results showed that the piR-000699 level was greater in the I/R group than in the sham group or in the H/R group ( Figure 3A,B). To further explore whether piR-000699 regulates ferroptosis-mediated processes in aging cardiomyocytes during H/R injury, we subsequently manipulated and overexpressed piR-000699 in these cells. As demonstrated in Figure 3C,D, successful establishment of a cell line was confirmed by measuring the expression levels of piR-000699. Notably, Figure 3E,F revealed significantly lower iron and MDA levels in the H/R+piR-000699 inhibitor group than in the H/R+inhibitor NC group. Conversely, iron and MDA levels were markedly greater in the H/R+piR-000699 mimics group than in the H/R+mimics NC group. Subsequently, we further evaluated the impact of piR-000699 on ferroptosis-mediated ageing-related myocardial ischaemia-reperfusion injury. As anticipated, the expressions of TFR and ACSL4 were significantly downregulated, while the expression of GPX4 (a prominent marker protein for ferroptosis) was markedly upregulated in the H/R+piR-000699 inhibitor group compared to the respective control group (H/R+ inhibitor NC) ( Figure 3G‒I). In contrast, the H/R+piR-000699 mimic group showed a significant increase in TFR and ACSL4 expressions relative to their respective controls (H/R+mimics NC), accompanied by a corresponding downregulation of GPX4 expression ( Figure 3J‒L). Moreover, the levels of the myocardial cell death markers CK-MB and LDH were significantly lower in the H/R+piR-000699 inhibitor group than in the H/R+inhibitor NC group ( Figure 3M,N). Conversely, the levels of these markers were noticeably greater in the H/R+piR-000699 mimic group than in the H/R+mimics NC group. Collectively, these findings confirm that piR-000699 regulates ferroptosis status and contributes to age-related myocardial I/R injury.

### piR-000699 targets *SLC39A14* in aging myocardial tissue

**Figure 4:**
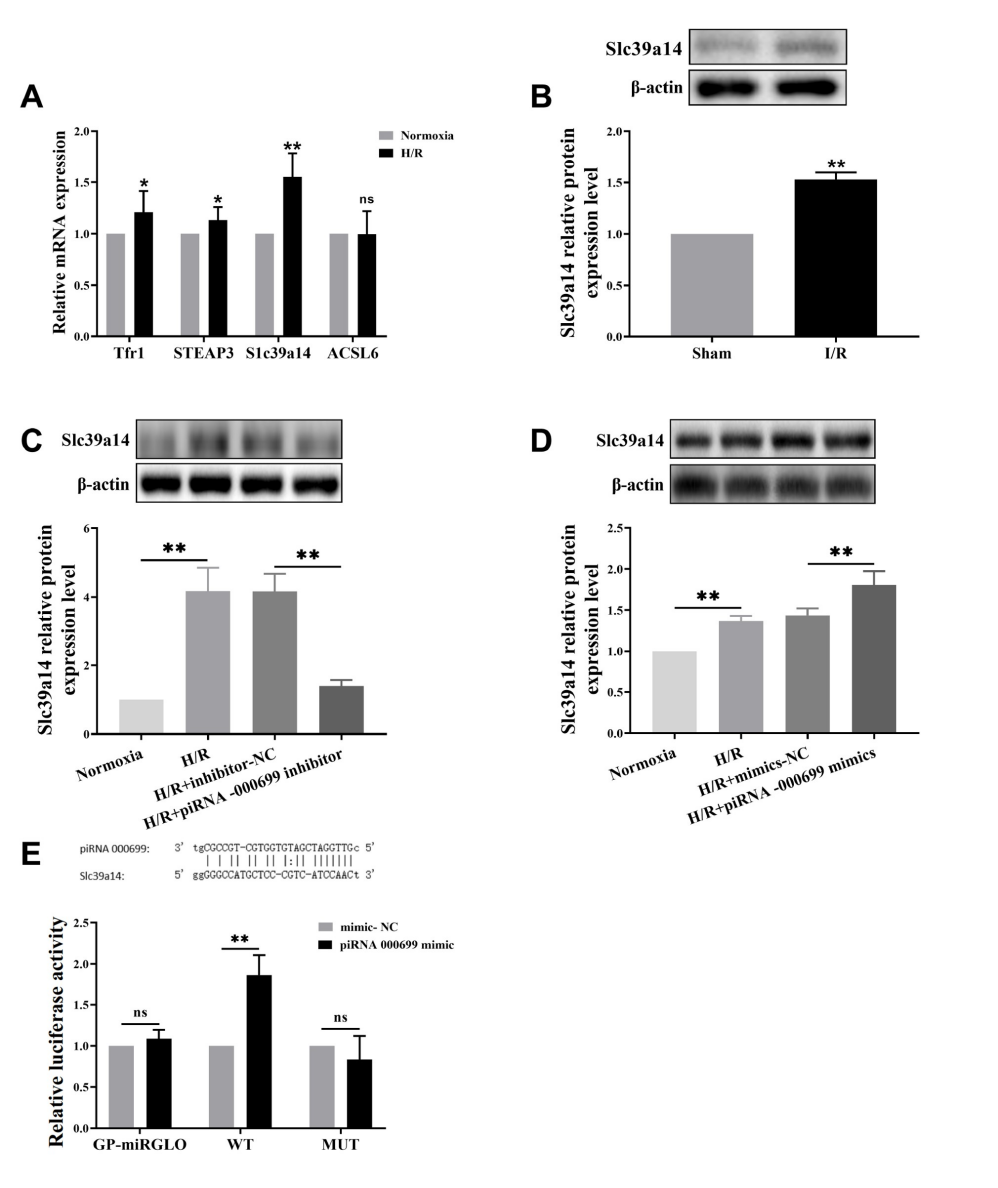
Figure 4 Screening and validation of piR-000699 downstream target gene *SLC39A14* in aging myocardial I/R injury (A) qRT-PCR was used to measure the expression of piR-000699 downstream target gene mRNA levels in H9C2 cells after the cells were subjected to H/R. (B) Western blot analysis was used to measure the protein expression of the piR-000699 downstream target gene SLC39A14 in the heart tissues of rats subjected to I/R surgery. (C) Western blot analysis was used to measure the protein expression of the piR-000699 downstream target gene SLC39A14 in H9C2 cells after they were subjected to H/R and piR-000699 inhibition. (D) Western blot analysis was used to measure the protein expression of the piR-000699 downstream target gene SLC39A14 in H9C2 cells after the cells were treated with H/R and piR-000699 mimics. (E) Targeting relationship between piR-000699 and SLC39A14 detected by a dual luciferase reporter gene system. The results are presented as the mean±SD. n=3‒5. * P<0.05, ** P<0.01 vs the normocia group / H/R+mimics NC group / H/R+inhibitor-NC group.

The mRNA expression levels of piR-000699 downstream target genes in H9C2 cells were quantified using qRT-PCR after treatment with H/R ( Figure 4A). Interestingly, *SLC39A14* mRNA exhibited a significant increase in the H/R group. Subsequently, western blot analysis was performed to measure SLC39A14 protein levels in rat heart tissues subjected to I/R surgery, revealing further exacerbation of this increase in the I/R group compared to the sham group ( Figure 4B). Notably, our results demonstrated that the SLC39A14 protein level was significantly lower in the H/R+piR-000699 inhibitor group than in the H/R+inhibitor NC group ( Figure 4C), while it was significantly greater in the H/R+piR-000699 mimics group than in the H/R+mimics NC group ( Figure 4D). To elucidate the molecular mechanism underlying this induction and determine whether there is a targeted binding relationship between piR-000699 and SLC39A14, luciferase activity assays were conducted on cotransfection groups consisting of piR-000699 mimics and WT constructs. Remarkably, luciferase activity was significantly greater in these cotransfection groups than in the mimic NC groups ( Figure 4E). In conclusion, our data suggest that piR-000699 exhibits targeted binding to its downstream target gene *SLC39A14* in aging myocardium.

### SLC39A14 attenuates aged myocardial I/R injury via ferroptosis

**Figure 5:**
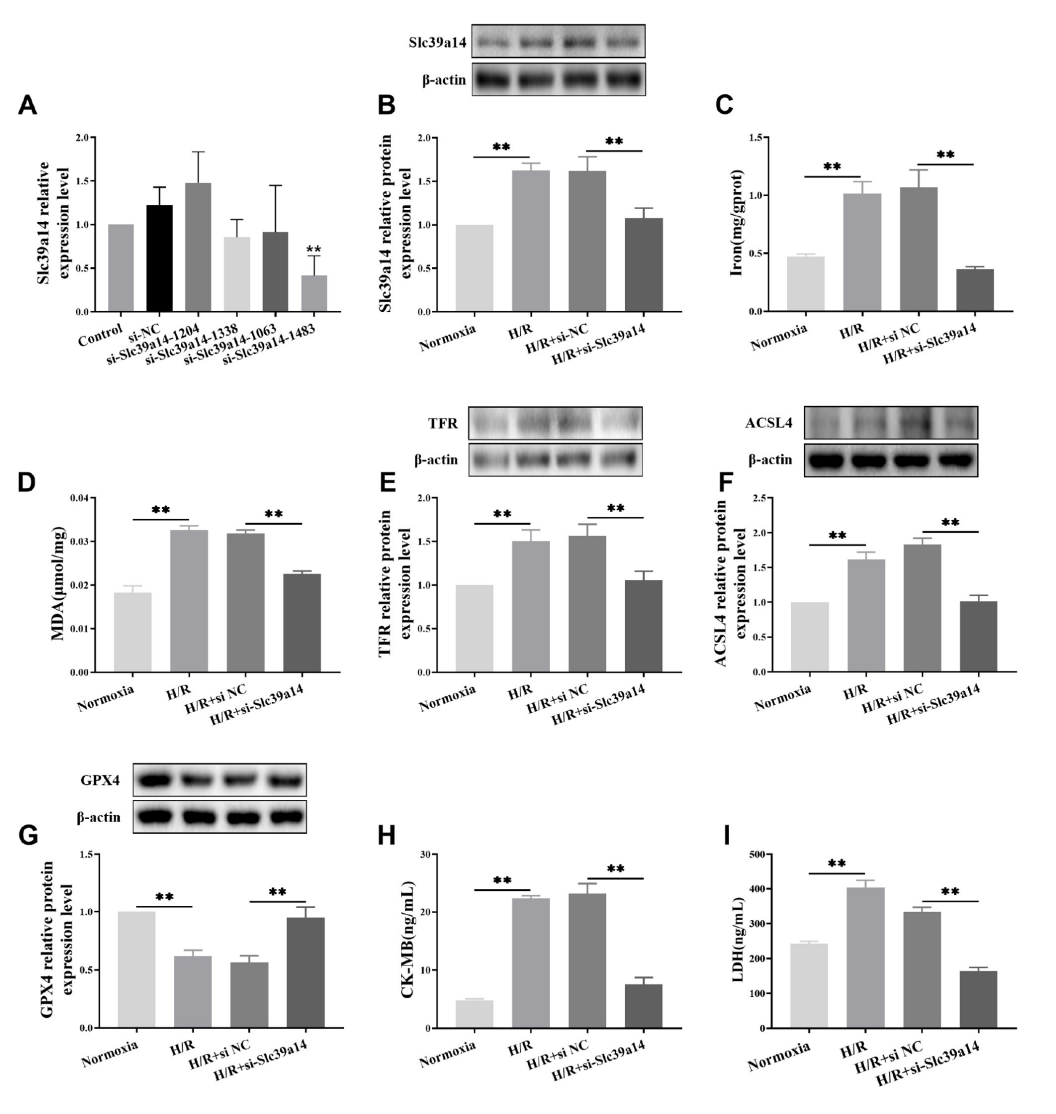
Figure 5 The effects of SLC39A14 on ferroptosis in aging myocardial ischemia/reperfusion injury (A) qRT-PCR was used to assess the effects of the SLC39A14 interference fragment. (B) Western blot analysis was used to measure the expression of SLC39A14 protein in H9C2 cells in which SLC39A14 was downregulated in the H/R group. (C) Iron concentrations in H9C2 cells in which SLC39A14 was downregulated in the H/R group were measured. (D) The levels of the lipid peroxidation product MDA in H9C2 cells in which SLC39A14 was downregulated in the H/R group were measured. (E‒G) Western blot analysis was used to measure the ferroptosis marker proteins TFR, ACSL4 and GPX4 in H9C2 cells in which SLC39A14 was downregulated in the H/R group. (H,I) Serum levels of the myocardial cell death markers CK-MB and LDH determined by ELISA in H9C2 cells in which SLC39A14 was downregulated in the H/R group. The results are presented as the mean±SD. n=3‒5. ** P<0.01 vs the Normocia group / H/R+si-NC group.

qRT-PCR revealed a reduction in the relative expression level of *SLC39A14* mRNA in the si-SLC39A14-1483 group compared to the si-NC group, indicating interference fragment screening ( Figure 5A). Furthermore, there was a noticeable decrease in SLC39A14 protein levels in the H/R+si-SLC39A14 group ( Figure 5B). As shown in Figure 5C,D, both iron and MDA levels were significantly lower in the H/R+si-SLC39A14 group than in the H/R+si-NC group. Western blot analysis demonstrated significant reductions in TFR and ACSL4 protein expressions, while GPX4 protein expression was notably increased in the H/R+si-SLC39A14 group ( Figure 5E‒G), indicating changes in ferroptosis marker proteins. Finally, the levels of the myocardial cell injury markers CK-MB and LDH were markedly reduced in the H/R+si-SLC39A14 group ( Figure 5H,I). These findings suggest that SLC39A14 plays a regulatory role in ferroptosis during aging myocardial I/R injury.

### piR-000699 targeting *SLC39A14* attenuates aged myocardial I/R injury through regulating ferroptosis

**Figure 6:**
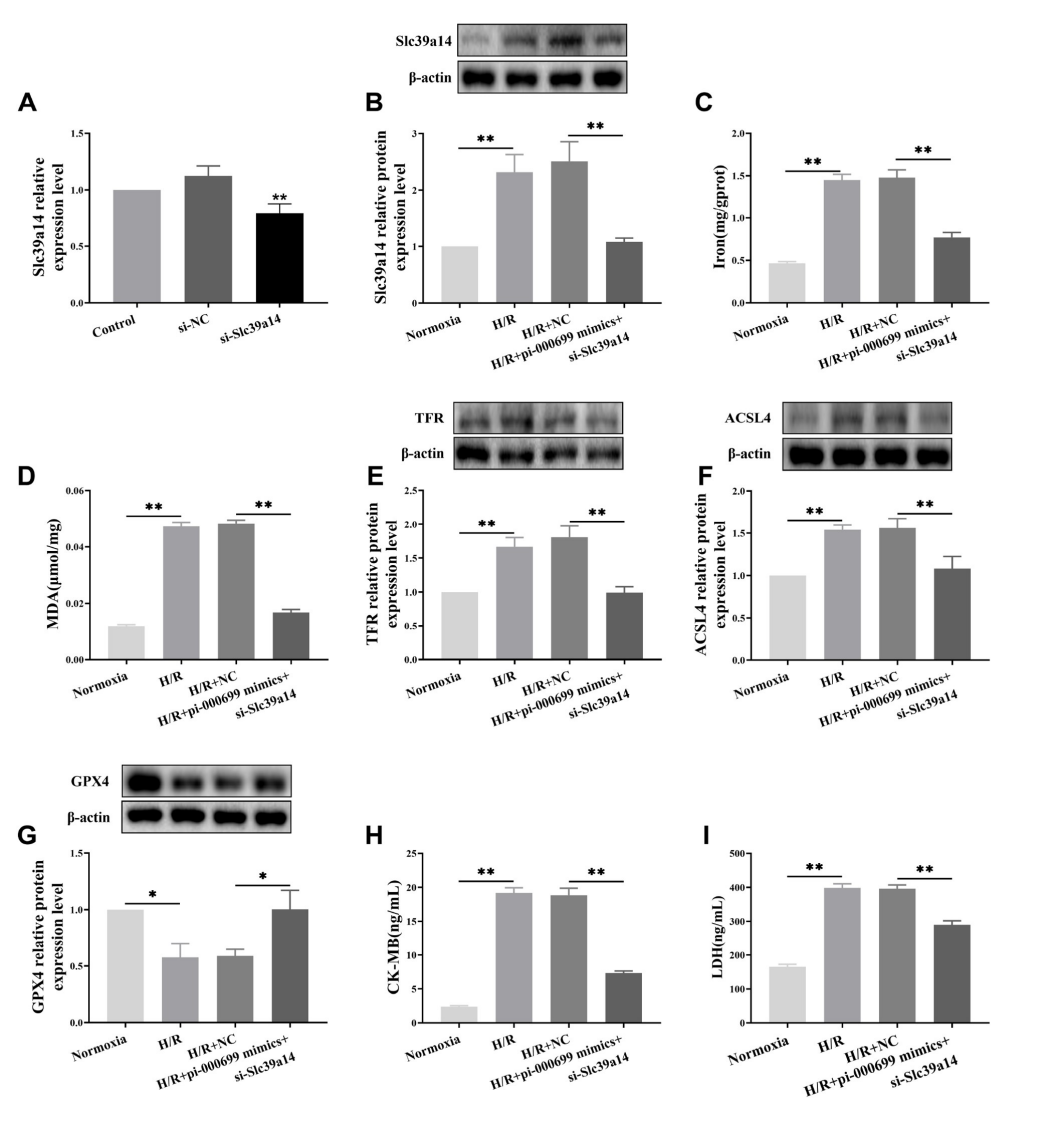
Figure 6 The effect of piR-000699-targeted binding to SLC39A14-regulated ferroptosis leading to aging myocardial I/R injury (A) qRT-PCR was used to measure the expression of SLC39A14 mRNA in the target gene inhibitor expression vector of aging myocardial cells. (B) Western blot analysis was used to measure the expression of SLC39A14 protein in aging myocardial cells transfected with the piR-000699 overexpression vector or the target gene inhibitor expression vector. (C) Iron concentrations were measured in aging myocardial cells transfected with the piR-000699 overexpression vector or the target gene inhibitor expression vector. (D) The levels of the lipid peroxidation products of MDA were measured after aging myocardial cells were transfected with the piR-000699 overexpression vector or the target gene inhibitor expression vector. (E‒G) Western blot analysis was used to measure the ferroptosis marker proteins TFR, ACSL4 and GPX4 in aging myocardial cells transfected with the piR-000699 overexpression vector or the target gene inhibitor expression vector. (H,I) Serum levels of the myocardial cell death markers CK-MB and LDH in aging myocardial cells transfected with the piR-000699 overexpression vector or target gene inhibitor expression vector determined by ELISA. The results are presented as the mean±SD. n =3‒5. * P<0.05, ** P<0.01 vs the normocia group / H/R+si-NC group.

To elucidate the underlying mechanism by which piR-000699 regulates ferroptosis in aging myocardial I/R injury, we successfully established a cell line with silenced *SLC39A14* expression. As shown in Figure 6A, the mRNA levels of *SLC39A14* were significantly reduced in the *SLC39A14*-silenced group. Subsequent investigations revealed that overexpression of piR-000699 and silencing of *SLC39A14* led to a significant decrease in the expression of the SLC39A14 protein ( Figure 6B). Moreover, when piR-000699 was overexpressed and *SLC39A14* was silenced, both iron and MDA levels were notably decreased within cells ( Figure 6C,D). Additionally, the protein expression levels of TFR and ACSL4 were significantly decreased ( Figure 6E,F), while GPX4 protein expression was markedly increased ( Figure 6G) under conditions in which piR-000699 was overexpressed and *SLC39A14* was silenced. Furthermore, in the H/R+piR-000699 mimics+si-SLC39A14 group, the levels of markers of myocardial cell death, such as CK-MB and LDH, were markedly reduced ( Figure 6H,I). In conclusion, our findings strongly suggest that piR-000699 regulates ferroptosis in aging myocardial I/R injury through targeted binding to SLC39A14.

## Discussion

Myocardial infarction mediated by I/R injury can induce irreversible aged myocardial cell loss and myocardial dysfunction, which are the main causes of morbidity and mortality in elderly patients [ 17, 18] . The prevention and control mechanisms of I/R injury in aged myocardial tissue have become a hot topic for scholars. Importantly, myocardial I/R injury further aggravates myocardial cell damage, causes myocardial cell apoptosis, and leads to a decrease in the number of viable myocardial cells [ 19, 20] ; therefore, it is extremely necessary to explore the mechanism of aged myocardial I/R injury in elderly individuals. In the present study, we established *in vivo* and *in vitro* models of aging-related myocardial I/R injury. We observed an increase in piR-000699 expression that correlated with ferroptosis indicators as well as the extent of myocardial cell damage. Moreover, our study aimed to elucidate how piR-000699, which targets *SLC39A14*, attenuates aged myocardial I/R injury through ferroptosis modulation—a finding that may contribute to early diagnostic methods and novel therapeutic strategies for reperfusion injuries associated with ischaemic cardiovascular diseases.

Ferroptosis is an innovative form of programmed cell death that relies on iron and iron deposition from apoptosis and necrosis [ 21, 22] . Ferroptosis can be activated by iron overload or by inactivation of GPX4, the major endogenous mechanism for preventing peroxidation 7–9, which converts potentially toxic lipid hydroperoxides into nontoxic lipid alcohols 10 [23]. A previous study showed that liproxstatin-1 (Lip-1) protected the mouse myocardium against I/R injury by suppressing ferroptosis through increasing levels of GPX4 [24]. Li *et al*. [25] indicated that the ferroptosis inhibitor Fer-1 also alleviated diabetes-induced myocardial damage in H9c2 cells in a high-glucose environment and prevented H9c2 cell damage during hypoxia/reoxygenation. In this study, we showed that ferroptosis indicators, such as iron content, TFR expression, and ACSL4 expression, were increased in an aging myocardial ischaemia‒reperfusion injury model; however, GPX4 expression was suppressed. Furthermore, there was a positive correlation between ferroptosis status and the concentrations of CK-MB and LDH. Therefore, inhibition of ferroptosis may represent a potential therapeutic option for treating or preventing myocardial I/R injury.

piRNAs, noncoding small RNAs that are primarily localized in the nucleus and form complexes with PIWI proteins [26], exhibit abundant expression in cardiac muscle tissue. The expression of these genes is also significantly altered during various stress conditions, such as myocardial infarction and hypertrophy [ 27, 28] . piRNAs also exhibit a dynamic and specific expression pattern during cardiac differentiation from pluripotent embryonic stem cells [29]. To date, the biological functions of piRNAs in the cardiac physiology and pathogenesis of various diseases remain largely unknown, especially in aging cardiomyocytes subjected to H/R injury. In this study, we showed that piR-000699 promotes cardiomyocyte dysfunction caused by aging myocardial ischaemia‒reperfusion injury through ferroptosis. Our results revealed that piR-000699 directly binds to SLC39A14 among its main downstream target proteins: TFR1, STEAP3, SLC39A14, and ACSL6. By constructing an inhibitor expression vector for target genes and transfecting it into senescent cardiomyocytes, we ultimately determined whether piR-000699 induces ferroptosis in these cells by targeting SLC39A14. This study focused on elucidating the regulation of ferroptosis by piR-000699 and investigated the role of combined targeting of piR-000699 and SLC39A14 in causing aging myocardial I/R injury. Our data suggest that targeted binding between piR-000699 and SLC39A14 regulates ferroptosis, leading to aging myocardial I/R injury. This study provides a theoretical basis for preventing and treating I/R injury.

This finding marked the inaugural exploration of the role of piR-000699 in ferroptosis-induced ischemic/reperfusion injury in aging myocardial cells, revealing pivotal targets and revealing a novel intervention strategy for targeted therapy against aging myocardial I/R injury. Nevertheless, several limitations exist within this study. Primarily, we conducted only *in vitro* experiments employing specific inhibitors or overexpression agents to investigate the underlying mechanisms involved. To further elucidate these mechanisms, our lab should use transgenic or knockout rat animal models or cells to overexpress piR-000699 and inhibit SLC39A14. Furthermore, this study merely encompasses animal and cellular level experimentation; thus, it necessitates validation through clinical trials before its application in humans can be considered viable. Despite these shortcomings, our study also offers a fresh perspective on the treatment of aging myocardial I/R injury.
